# Perception of the Medical Students on Their Future Career in Qazvin University of Medical Sciences

**DOI:** 10.5539/gjhs.v4n4p176

**Published:** 2012-06-25

**Authors:** Ameneh Barikani, Mahsa Afaghi, Firooze Barikani, Ahmad Afaghi

**Affiliations:** 1 Department of Community Medicine, Qazvin University of Medical Sciences, Qazvin, Iran; 2 Western Sydney University, New South Wales, Australia; 3 Alborz Educational Center; 4 Metabolic Diseases Research Center, Qazvin University of Medical Sciences, Qazvin, Iran

**Keywords:** attitude, medical students, future career, general practitioner

## Abstract

**Introduction:**

Young physicians have many recruitment barriers in Iran. Therefore, for planning purpose, assessment of the attitudes of medical intern students towards their future career is important.

**Methods:**

This cross-sectional study assessed the view points of 300 medical students through a self administered questionnaire. Data were analyzed using SPSS software with P value < 0.05.

**Results:**

Two hundred and forty students (80%) of the students had responded to the questionnaire. Among them, 67.5% were female with mean age of 21.7±2.4. The main factors for deciding to study in medicine were their interest (64.1%), family pressure (13.5%) and social prestige of medical career (9.8%). The mean score of attitudes was 2.3±0.6. In total, 24.5% of students demonstrated not having interest in studying medicine. The most important cause of their interest change was long duration of education (24.4%) and cost of studying in medicine (13.8%). In total, 88.6% of students had negative viewpoint towards their medical career in future.

**Conclusion:**

In general, the attitude of medical students toward their future career was negative.

## 1. Introduction

The imbalance between the public needs and the number of the medical graduates has created several problems in recruitment of young physicians who are trained to provide medicaal and health services in the country. There were six medical schools in the academic year of 1965-1966 in Iran and just before the revolution in 1979; this number had risen to 13. There were 48 medical schools educating physicians in year 2006 (Asefzadeh et al., 2002; Mohamadi & Mojtahezadeh, 2003). In last two decades, the policy of ministry of health in Iran, was to increase medical students’ admission to overcome health and medical needs of fast populated country. In year 2008, there was one medical doctor for every 690 persons in Iran (Powis, 2003).

Although the increased number of medical schools and medical students overcame shortage of physician in Iran and all rural areas benefited from a health care system, but young physicians faced to frequent problems such as, unsupportive learning environment, limitation to the evidence based medical skill, lack of awareness of research, low income, job insecurity and consequently reduced interest to work in health system, especially in rural area (MHTME, 1992). There are limited studies in relation to unemployment and income of physicians in Iran. In a report of management and planning office in year 2005, 12.2% of General Practitioners (GP) were unemployed (MHTME, 1992). And several studies showed that 13.5% of GP are working in unprofessional jobs (Salehzadeh & Hassanzadeh, 1999). In Iran, health care system expect physician to practice as medical expertise and also to work as both health care resource manager and socially aware healer. Therefore, the expectation from physician is far from their potential (Niemi et al., 2003).

In viewpoints assessment of internship students at Qazvin University of Medical Science in year 2002, 89.2% of students expressed that the present system of medical education does not give them the necessary capabilities and skills to practice efficiently in the future (Asefzadeh et al., 2002). In another study, 67% of medical students even in primary stages of education didn’t have any interest to work in villages and rural area. Theses studies show that, although, the aim of ministry of health in increasing number of medical students was compensating of shortage of GP in health system, but their strategy was not successful (Amini & Rezayi, 2004).

Therefore, this study aimed to determine attitudes of all medical students’ in different educational stages, towards their future careers.

## 2. Methods

This survey was conducted in 2008-2009 to obtain medical students viewpoints at Qazvin University of Medical Science through a self administered questionnaire. The questionnaire was designed by the researchers and validated by a medical education expert, an epidemiologist and social medicine specialist. A pretest on a random sample carried out to ensure that the questions were clear. The questionnaire consisted of two parts; the first section included demographic data and in second section students’ viewpoints on different aspects of medicine and their future career was sought.

### 2.1 Participant

Three hundred medical students studying at Qazvin University of Medical Science were recruited, the students answered anonymously and their participation was optional. The first 5 semeters of medical education in Iran includes basic science, the second 4 semesters, is physiopathology, the third 3 semesters is clerkship and fourth phase of education is internship. The Likert scales were checked on a 5-point scale from “very good” to “very poor” (very good=1, good=2, moderate=3, poor=4, very poor=5) to assess the subjects’ viewpoint. Answers were classified into positive or negative according to the prevalence of responses. If the frequency of “very good” and “good” responses were more than the “very poor” and “poor” ones, the viewpoint was considered positive.

### 2.2 Statistics

Frequency, mean and standard deviation of attitudes were computed. ANOVA were performed to detect differences in attitudes among 4 education stages (basic science, physiology, clerkship and internship)

The t-test was used to compare answers to various questions among 4 groups. A two-sided p-value of 5% was considered significant for all statistics.

## 3. Results

Two hundred and thirty nine (80%) of students filled out the questionnaire. From all respondents, 67.5% were female. Mean age of participants was 21.7±2.4, 42% of subjects were in basic science, 12.6% in physiopathology, 24.5% in clerkship and 20.6% in internship stage. The main factors for decision to study in medicine are summarized in Table 1. Interest of medical students towards their selection was increased by 13.9% (Table 2).

**Table 1:** The frequency of distribution of the main factors for decision to study medicine at Qazvin University of Medical Science (N=239)

My interest	64.11
Social position of medical career	9.8
Scientific position of medical career	5.4
Economic position of medical career	2.5
Family pressure	13.5

**Table 2:** The change of medical student’s interest towards their selection by stage of education at Qazvin University of Medical Science (N=239)

Stages	increased	decreased	Non-change	Total
basic	13.5	10.2	18.2	42
physiopathology	4.6	3.4	4.5	12.6
clerkship	5.5	7.6	11.7	24.8
internship	7.6	3.3	9.7	20.6
total	31.2	24.5	44.3	100

Data are expressed by percentage

In response to the question “to what extent can a medical career meet your economic needs”, 11.4% and 40.7% chose “very poor” and “poor” respectively (Table 4) and 88.6% of students had negative viewpoint towards medical career. In response to the question “what is your decision about your future career after graduating”, 61.4% had answered to continue their studies in specialty of medical field and 21.8% mentioned to continue their studies abroad. Around 1.7% of students had decided to work as a General Practitioner, 2.4% had chosen non-medical careers and 12.7% didn’t have any idea. The mean score of attitude was 3.2±0.6 and there was no significant difference between stage of education and sex, but significant difference was observed between two sexes towards financial (p<0.001) and job security (p<0.02). In addition significant difference between four groups of educators toward satisfaction of the moral and emotional needs (p<0.009), and opportunity to work in a scientific field (p<0.001) was observed.

**Table 3:** The cause of attitude change among medical students at Qazvin University of Medical Science (N=239)

Long duration of education	24.4
Low financial	13.8
Low job security	8.8
Change of public attitude	7.2
Total	45.8

Data are expressed by percentage

**Table 4:** The students viewpoints on aspects of medical professions at Qazvin University of Medical Science (N=239)

	Very good	good	Moderate	poor	Very poor
Financial security	1.7	5.9	40.3	40.7	11.4
Self welfare and comfort	6.3	20.8	36.4	26.3	10.4
Job security	6.4	16.1	41.1	29.2	7.2
Satisfaction of the moral and emotional needs	3.4	8.5	31.8	36.8	19.5
Opportunity to work in a scientific field	4.3	10.3	30.2	28.7	26.6
The scientific value of medicine in the future	0.4	6.3	28.6	33.2	31.1
The social value of medicine in the future	1.7	2.6	31.2	39.2	25.4

Data are expressed by percentage

**Table 5:** Mean score of attitude in female and male medical students at Qazvin of University of Medical Science

Factor	female	male	P
Financial security	34±0.8	3.7±0.8	0.01
Self welfare and comfort	3±1	3.2±1	0.17
Job security	3±0.4	3.3±0.9	0.02
Satisfaction of the moral and emotional needs	3.5±0.9	3.6±1	0.63
Opportunity to work in a scientific field	3.5±1	3.7±1	0.30
The scientific value of medicine in the future	3.8±0.9	3.8±0.8	0.79
Social value of medicine in the future	3.8±0.9	3.9±0.9	0.81

**Table 6:** Mean score of attitude in different stage of medical students at Qazvin University of Medical Science

View	basic	physiopathology	clerkship	internship	p
Financial security	3.5±0.8	3.2±0.4	3.6±0.7	3.5±0.8	0.2
Self welfare and comfort	3±1	3.3±0.11	3.9±0.9	3.4±1.2	0.5
Job security	3.1±0.9	2.9±1	3.1±0.8	3.2±1	0.5
Satisfaction of the moral and emotional needs	3.7±1	3.4±10	3.3±0.9	3.8±0.7	0.009
Opportunity to work in a scientific field	3.8±0.9	2.9±1	3.3±1	4±0.9	0.000
Social aspect and public view	4.1±0.8	3.3±0.9	3.6±0.8	4±0.9	0.000
The scientific value of medicine in the future	3.9±0.3	3.6±1	3.7±0.7	3.8±0.1	0.3

## 4. Discussion

Our data showed that, the most of medical students, had negative attitude toward medical career and most of them believed that medical profession doesn’t have the potential to provide welfare and comfort. In previous studies conducted in Iran, it was shown that, 60% of students’ attitude toward medical study and future career was changed to negative (Sadr, 1999; Hajian & Nasiri, 2006). These attitude changes had happened in the last years of education when the students face with reality in the community. In early stages of education program medical students due to succeed in entering to medical university and perception of having good job position in future are happy and satisfied, but they have not right understand of problems in this profession.

Students who select medicine for future career with own interest and without pressure or other factors such as social view or economic aspects, have more positive view and their attitude would be changed less. In two studies by Donnelly and watte in UK and USA students satisfaction was high (Donnelly et al., 1996; Watt et al., 2005; Hajian & Nasiri, 2006).

Although in our study the majority of students, based on their own interest, had applied to study medicine (64.1%), but other factors such as social, scientific, economic position of medical career, and family pressure had significant role. Intention to enter medical university is ideal for any school students and their families in Iran. However, when the students enter the medical university, they exposed with reality and many problems such as anxiety about aspects of their future career. School students usually enter to medical university in young age, therefore they haven’t sufficient familiarity to problems of this profession and they were not prepared to this profession. In a study carried out in Iran, in respond to qualitative question towards cause of choosing medicine for educating, the isues such as family pressure and social position were main factors, but in respond to quantitative questions scientific interest was main factor (Nejat et al., 2006).

One study conducted in Iran revealed that, 15% of GPs were working in un-medical profession and 5% were abroad (Niemi et al., 2003). Unemployment in medical graduates was not formally announced, but in different reports it was around 12.2%. In our study job and financial security of medical profession in view of female students was more negative than males, although men are responsible for living cost in Iran, but responsibilities of females are growing rapidly in addition to their other duties at home. This results in more anxiety among females, causing them to have more negative views about their attitude, financial security, self-welfare, comfort and opportunity to work in a scientific field. In several studies sex is one of predicting factor for job motivation (Cujec et al., 2000; McMurray et al., 2000; Finset et al., 2005; Hajian & Nasiri, 2006).

Long duration of education, unemployment and economic problems could also change students’ expectations that if they don’t have hope for meeting, could lead to negative attitude. In other hands, tend to continue of education after GP in Iranian physicians is very high (Javadi & Asefzadeh, 2000) and they don’t like to work long duration as GPs in health system. This leads to proloveralnged education period and increases their expectations. The most of GPs that are graduated from medical university must spend a 2-year obligatory service in ministry of health in remote area.

In our study 85% of the students believed that the present medical education system does not provide necessary skills for their future career. The medical education system has remained rigid and unchanged for years and a revision based on occupational needs seems necessary. There is a serious need to move forward with educational reform in order to provide communities with the doctors they need. Almost all Iranian medical schools are still offering course based on the traditional system that follows a discipline based approach, teacher centered and hospital based.

Communication skills training course have no place within the current undergraduate medical curriculum. In Iran there is large evidence that indicate, the importance of medical students’ communication skills. According to the AMA potential physicians, need motivation and intelligence, evidence of leadership and good communication skills.

In conclusion authors suggest that policy makers must notice more towards physicians employment and their social and economical welfare. Education centers must inform medical students about medical training and practice before they had entered the medical school. The teaching patterns must be revised in order to train graduates that become familiar with realistic problems in the community.
